# Histopathologic findings on removed stomach after sleeve gastrectomy. Do they influence the outcome?

**DOI:** 10.1515/med-2022-0450

**Published:** 2022-03-09

**Authors:** Giovanni Tomasicchio, Arcangelo Picciariello, Rigers Dibra, Giuliano Lantone, Giuseppe Trigiante, Michele De Fazio, Gennaro Martines

**Affiliations:** Department of Emergency and Organ Transplantation (DETO), University of Bari Aldo Moro, Piazza Giulio Cesare, 11, Bari, Puglia, Italy

**Keywords:** laparoscopic sleeve gastrectomy, histopathologic findings, excess weight loss, excess BMI loss, *Helicobacter pylori* infection

## Abstract

Little is known about the role of chronic gastritis on weight loss after laparoscopic sleeve gastrectomy (LSG). This study aims to investigate the relationship between histopathologic findings of gastric specimens, excess weight loss (% EWL), and excess BMI loss (% EBL) at 6 and 12 months follow up after LSG. We retrospectively reviewed the clinical records of 95 patients who had undergone LSG between January 2017 and December 2019. Based on the histopathological findings of gastric resection specimens, patients were divided into those with chronic gastritis (CG) and those without chronic gastritis (NoCG) and compared for their % EWL and % EBL at 6 and 12 months. The mean BMI was 44.74 kg/m^2^ in the CG group and 44.14 kg/m^2^ in the NoCG group. At 6 months follow up, the CG group had a mean % EWL of 45.7 and % EBL of 40.5, while NoCG had a mean % EWL of 51.1 and % EBL of 46.7. After 1-year follow-up, the CG group had a mean % EWL of 53.1 and a % EBL of 44.8, while the NoCG group had a % EWL of 54.1 and % EBL of 44. This observational study does not support the hypothesis that the occurrence of chronic gastritis can affect postoperative % EWL and % EBL.

## 1 Introduction

The prevalence of obesity worldwide has approximately doubled since 1980 and over one-third of the world’s population is estimated to be overweight or obese [1]. Lifestyle changes, physical activity, diet, and medical therapies seem to be ineffective in the treatment of severely obese patients (BMI > 40 kg/m^2^) or in morbidly obese with comorbidities [2]. To date, bariatric surgery is the most effective curative treatment [3]. Laparoscopic sleeve gastrectomy (LSG) is the most frequent bariatric surgical option in Italy, thanks to its relative low morbidity and mortality and for the satisfactory results in weight loss in the long-term [4]. However, even after LSG, some patients fail to achieve adequate weight loss for unknown reasons. Surgical/anatomic factors, hormonal/metabolic imbalance, and behavioral/mood factors have been shown to be able to affect weight loss following LSG [5]. However, other possible influencing factors include histopathologic abnormalities of the stomach, which can affect the production/release of ghrelin and other enterohormones.

Patients undergoing LGS are presumed to have no significant gastric diseases, and, therefore, routine microscopic examination of LSG specimens has been suggested to be unnecessary [6]. At the same time, several studies demonstrate unexpected microscopic alterations in sleeve gastrectomy specimens, such as intestinal metaplasia (IM), *Helicobacter pylori* (HP) infection, gastrointestinal stromal tumor, follicular gastritis, lymphoid aggregates, and chronic active and inactive superficial gastritis [2,7,8]. Other studies investigated the role of chronic gastritis, with or without HP infection, on the secretory activity and density of gastric ghrelin cells and how they regulate eating behavior and weight balance, leading to controversial results [9,10].

This retrospective observational single-center study aims to investigate the relationship between the presence of chronic gastritis, excess weight loss percentages (% EWL), and excess BMI loss percentages (% EBL) at 6 and 12 months postoperatively.

## 2 Materials and methods

Patients affected by severe obesity (BMI > 40 kg/m^2^) subjected to LSG in our surgical unit between January 2017 and December 2019 were enrolled in the study. Patients were followed up at 6 and 12 months postoperatively by clinical examination, serological tests, and esophagogastroduodenoscopy (EGD). Only those completing 1-year follow-up were included in the study. Demographic data including age, gender, weight (kg), BMI (kg/m^2^), % EWL, % EBL, and comorbidities (cardiovascular disease: hypertension, arrhythmias, coronary artery disease, and heart valve complication. Respiratory disease: Chronic obstructive pulmonary disease, asthma, and chronic bronchitis. Osteoarthritis: degenerative arthritis and degenerative joint disease.), were prospectively recorded. In the preoperative work-up, all patients underwent routine EGD in accordance with the European Association for Endoscopic Surgery and Italian Society of Bariatric Surgery recommendations [11]. Before surgery, all patients were given a rapid urease test for HP, and those with positive test result were treated with “*Pylera” (Allergan Pharmaceuticals International Ltd Ireland),* an association of bismuth subcitrate potassium, metronidazole, and tetracycline, 12 tablets a day for 10 days, associated with pantoprazole 40 mg 1 tablet a day. HP eradication was confirmed by a further rapid urease test. Patients without HP eradication, at the end of the therapy, were excluded from the study.

According to the histopathology of the gastric specimens, patients were divided as those with and without chronic gastritis.

The occurrence or not of chronic gastritis (CG) on the gastric specimens was correlated with preoperative BMI, % EWL and % EBL at 6 and 12 months after surgery. Furthermore, HP re-infection was checked post-operatively at 12-months follow-up by endoscopic biopsies and/or Urea Breath test.

This study was approved by our institutional review board and informed consent was obtained from all patients before enrolment. All investigations complied with the principles of the Declaration of Helsinki.

## 3 Statistical analysis

Continuous parameters were reported as median and interquartile ranges. Categorical variables were recorded as numbers and percentages wherever appropriate. Comparisons of categorical variables were performed by the Chi-square and Fisher’s Exact test wherever appropriate. Comparisons between groups were made using the Mann–Whitney *U* test. A *p* value < 0.05 was considered statistically significant. Statistical analysis was carried out using RStudio (R version 4.0.3 (2020-10-10) Copyright (C) 2020 The R Foundation for Statistical Computing).

## 4 Results

Ninety-five patients, 82 females and 13 males, entered the study. Median age was 43 (37–52) years. All patients were affected by severe obesity with a median BMI of 44.62 (40.80–47.75) kg/m^2^. Fifty-eight of them (61%) had histological evidence of chronic gastritis on the gastric specimen (median age 43 (36–52) years, 86% females). The remaining 37 (39%) patients had no signs of chronic gastritis (median age 43 (39–50) years, 86% females): 33 had no alteration of the specimens, 3 had IM, and 1 patient had muscular hyperplasia. Similar distribution of comorbidities was found between the CG group and NoCG group: cardiovascular 35 (60%) vs 25 (68%) and *p* = 0.621, diabetes 18 (31%) vs 13 (35%) and *p* = 0.848, arthropathy 11 (19%) vs 10 (27%) and *p* = 0.503, and respiratory 40 (69%) vs 20 (54%) and *p* = 0.210. There were no significant differences between the two groups concerning the distribution of comorbidities or presence of at least one comorbidity: none 11 (19%) vs 4 (11%), and with at least one comorbidity 47 (81%) vs 33 (89%) and *p* = 0.390 Table 1.

**Table 1:** Relationship between demographic features and comorbidities of CG and NoCG groups

	Total	CG	NoCG	*p* value
	*n* = 95	*n* = 58	*n* = 37	
Age (year)	43 *(37–52)*	43 (36–51.7)	43 (39–50)	*0.942*
Gender (M/F)	13/82	8/50	5/32	*1*
BMI (kg/m^2^)	44.62 *(40.80–47.75)*	44.7 (40.8–47.6)	44.1 (41.0–48.3)	0.619
**Comorbidity**				
None	15	11 (19%)	4 (11%)	
At least one comorbidity	80	47 (81%)	33 (89%)	*0.390*
Cardiovascular	60	35 (60%)	25 (68%)	*0.621*
Diabetes	31	18 (31%)	13 (35%)	*0.848*
Arthropathy	21	11 (19%)	10 (27%)	*0.503*
Respiratory	60	40 (69%)	20 (54%)	*0.210*

Note: *p* value > 0.05 was considered statistically significant.

Thirty-eight patients (40%) had a pre-operative HP infection which was successfully cured by medical treatment and were therefore included in the study.

**Figure 1:**
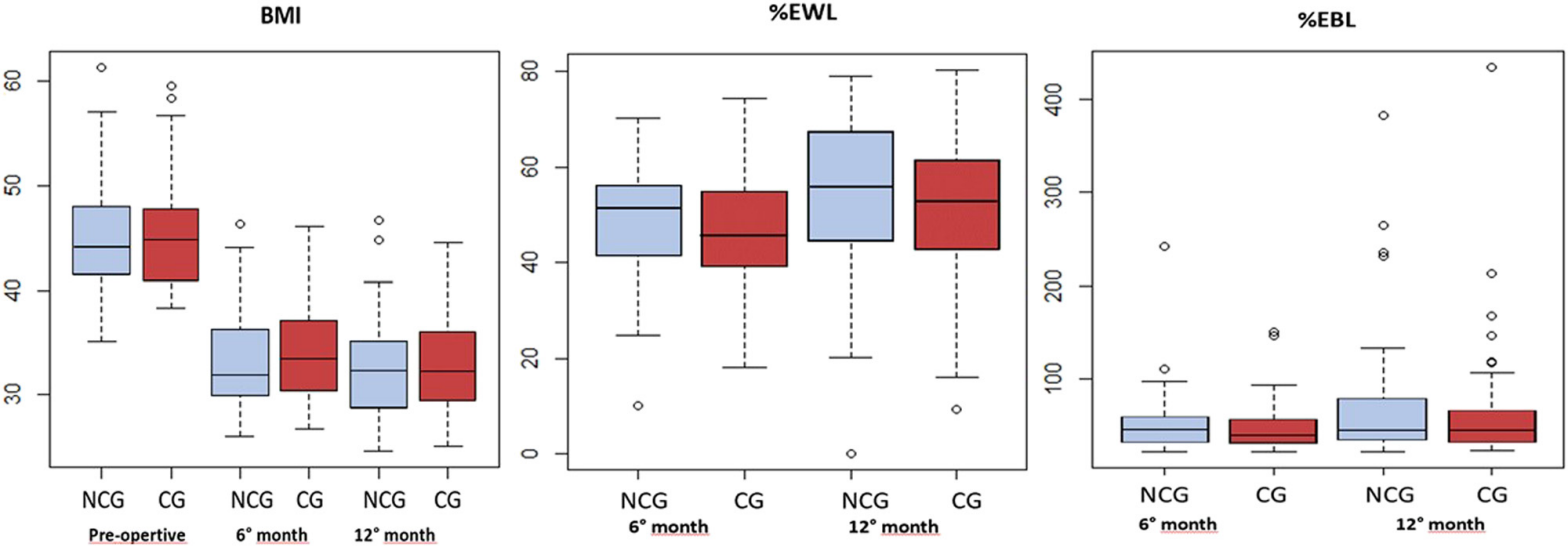
The relationship between BMI, % EWL, and % EBL at 6, 12 months in patients with CG (CG, *n* = 58) and without CG (NoCG, *n* = 37) on the specimens of sleeve gastrectomy.

The relationship between histopathologic findings (HF) and BMI, % EWL, % EBL at 6 and 12 months is shown in Figure 1. No significant difference was recorded in terms of preoperative BMI among patients with or without chronic gastritis. At 6 months, the CG group had a higher BMI and lower EWL/EBL when compared to the NoCG group (BMI 33.19 kg/m^2^ vs 31.8 kg/m^2^, % EWL 45.78 vs 51.1, % EBL 40.52 vs 46.76, respectively), although these differences did not reach statistical significance. Furthermore, no significant differences were found at 12 months between the 2 groups Table 2.

**Table 2:** Relationship between BMI, % EWL, and % EBL at pre, 6th, and 12th month in CG and NoCG

	Preoperative	6th month			12th month		
	BMI	BMI	% EWL	% EBL	BMI	% EWL	% EBL
**Chronic gastritis**							
Yes (*n* = 58)	44.7 (40.8–47.6)	33.1 (30.4–36.9)	45.7 (38.6–54.7)	40.5 (30.8–56)	32.1 (29.3–35.9)	53.1 (42.7–62.3)	44.8 (32.8–67.2)
No (*n* = 37)	44.1 (41.0–48.3)	31.8 (29.9–36.2)	51.1 (41–56.9)	46.7 (32.2–60.9)	32.2 (28.5–35.4)	54.1 (44–67.9)	44 (33–76.3)
*p* value	0.619	0.538	0.536	0.538	0.416	0.516	0.711

At 12^-^months follow-up, all patients underwent a rapid urease test or/and endoscopic biopsies to test the possible occurrence of a new HP infection and 15 of them (16%) tested positive. Nine of these (60%) belonged to the chronic gastritis group and 6 (40%) to the group without chronic gastritis. Two of the 15 patients with positive HP test were already positive before surgery (reinfection), while the remaining 13 (87%) patients had the infection for the first time.

**Figure 2:**
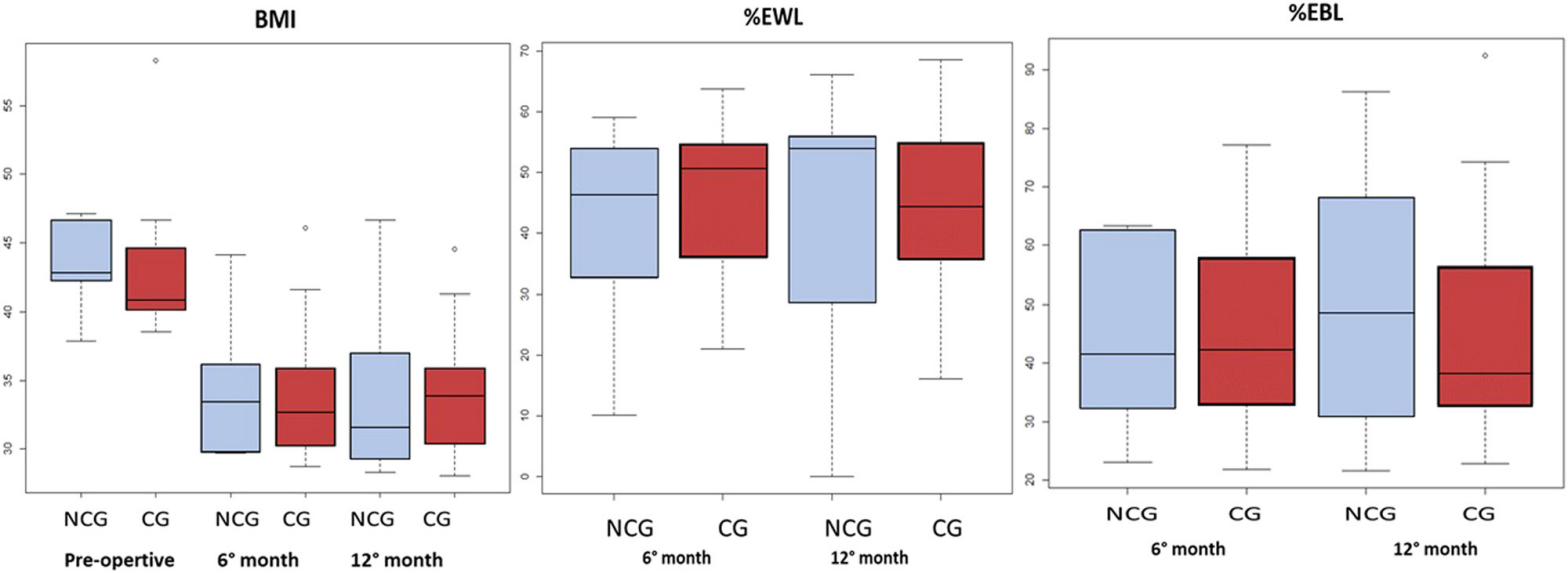
The relationship between BMI, % EWL, and % EBL at 6 and 12 months in patients with further HP infection with CG (CG, *n* = 9) and without CG (NoCG, *n* = 6) on the specimens of sleeve gastrectomy.

Figure 2 shows the relationship between BMI, % EWL, and % EBL at 6 and 12 months in patients with further HP infection with no significant differences recorded between the groups concerning BMI, % EWL, and % EBL. However, at 12 months after the surgery, the CG group had a greater BMI and lower EWL/EBL when compared with NoCG group (BMI 33.83 kg/m^2^ vs 31.5 kg/m^2^, % EWL 44.4 vs 53.9, and % EBL 38.3 vs 48.5, respectively) although this data did not reach a statistical significance Table 3.

**Table 3:** Relationship between BMI, % EWL, and % EBL at pre, 6^th^, and 12th month in CG and NoCG in patients with further HP infection

	Pre-operative	6th month			12th month		
	BMI	BMI	% EWL	% EBL	BMI	% EWL	% EBL
**Further HP infection**							
Chronic gastritis (*n* = 9)	40.7 (40.1–44.6)	32.7 (30.2–35.9)	50.7 (35.9–54.7)	42.3 (32.8–57.8)	33.8 (30.3–35.9)	44.4 (35.8–54.9)	38.3 (32.8–56.3)
No Chronic gastritis (*n* = 6)	42.7 (42.2–45.7)	33.4 (30.1–36)	46.3 (35.2–53)	41.70 (32.4–59.5)	31.5 (29.6–35.8)	53.9 (34.7–55.9)	48.5 (34.1–64.3)
*p* value	0.528	1	0.688	1	0.95	0.775	0.954

## 5 Discussion

LSG has gained popularity among bariatric surgeons because of its low morbidity and mortality and for its effectiveness in promoting patients’ weight loss. Nevertheless, a percentage of patients did not lose weight for unknown reasons.

The relationship between chronic gastritis, HP infection, and outcome of LSG in obese patients is still a controversial issue. Chronic inflammation of the stomach has been demonstrated to be able to induce changes in the gastric mucosa involving the density and secretory activity of gastric ghrelin cells, with abnormal regulation of eating behavior and weight balance following LSG [9]. In fact, plasmatic concentration of ghrelin has an important role in weight loss [12]. Ghrelin leads to an increase in appetite, energy intake, and inhibition of Leptin secretion. The resection of the gastric fundus, where ghrelin-producing cells are mostly represented, leads to a reduction in its plasma level [13,14]. In patients with chronic gastritis, several studies demonstrated an increase in circulating acylated ghrelin, hypothesizing that there may be a compensatory process to stimulate gastric acid production, since chronic gastritis leads to loss of ghrelin-producing cells and an increase in gastric pH [15,16]. Another study hypothesized that chronic gastritis (HP and no-HP related) can influence glycemic control with a negative effect and increases gastric emptying time [17]. Based on these considerations, Erkinuresin et al. suggested that hormonal feedback mechanisms in patients with CG could lead to a failure of the effects of LSG in reducing BMI [18].

In our study, histopathologic examination of resected gastric specimens revealed a significant percentage (61%) of patients affected by chronic gastritis despite the negative preoperative gastroscopy. These findings are in agreement with other studies focused on the pathologic findings of specimens after sleeve gastrectomy [8,18,19,20], further supporting the hypotheses that CG may play an important role in the development of severe obesity [9,20].

In our study, the group with CG had lower % EWL and % EBL and higher BMI at 6 and 12 months with respect to the NoCG group; however these differences did not reach a statistical significance.

These data are in line with Saafan et al. [8] who, in their series of 1,555 patients, found no significant association between preoperative BMI and histopathological findings in gastric specimens, after controlling for confounding variables (age, gender, HP, diabetes type 2, and hypertension). Likewise, Adali et al. [21] reported no significant difference between preoperative BMI and the presence or absence of gastritis or HP infection, while a significant difference was found between BMI and IM.

In fact, Kim et al. showed that patients with IM had lower levels of plasma ghrelin, compared to patients with other gastric histopathological alterations [22]. Erkinuresin et al. on the other hand found that IM had a negative effect in % EWL and % EBL with a difference of more than 10 points at 12 months compared to patients without IM, speculating that other mechanisms favoring obesity may occur in these patients [18]. In our series, we only found 3 patients with IM in LSG specimens, which prevented us from making any assumptions on this topic.

The effect of HP colonization on gastric mucosa in the regulation of food intake and BMI is controversial. This gram-negative bacterium infects approximately 4.4 billion people worldwide [23,24]. Several authors investigated the relationship between HP infection and hormonal modulation of food intake, with controversial results.

In our study all patients underwent a preoperative rapid urease test for HP, and positive patients underwent medical treatment to eradicate the infection. Nevertheless, 15 patients (16%) developed a new HP infection at 12-months follow-up. However, most of them developed the HP infection for the first time, while only 5% of them had a recurrence of the infection, in agreement with literature [25].

The *H. pylori*-induced inflammatory response of the gastric mucosa involves several gastric cell types, playing a role in the regulation of ghrelin and leptin production [26]. Leptin is a polypeptide hormone secreted by adipose tissue proportional to body fat content. It acts to reduce hunger, increases metabolic rate and thermogenesis by activation of the JACK-STAT pathway in the hypothalamus [27]. The plasmatic concentration of this hormone increases after food intake to control body weight. Mantero et al., in their cross-sectional study, showed low serum ghrelin concentration and no association with leptin serum levels in patients with HP infection compared with healthy controls, irrespective of their BMI or gender [10]. Roper et al. instead found that circulating leptin levels were significantly lower in patients with HP infection, hypothesizing a possible role in the modulation of the gastric cytokine function, altering the balance of leptin release into the luminal and systematic compartments. They also suggested that HP colonization could inhibit gastric leptin production by inducing atrophic changes in leptin-producing tissues. At the same time, they did not find an effect of HP status on circulating ghrelin levels, suggesting that the long-term carriage effect of HP on ghrelin production is minimal due to host adaptation [28]. Zhang et al., in their series of 39,091 individuals, found no significant difference in BMI changes between patients who had an eradicated HP infection and those with persistent infection or new to HP infection [29].

In our study, there were no significant differences between groups with reoccurring HP infection. The unexpected high rate of HP reinfection and higher BMI in patients with HP infection at 12-month postoperative follow-up should underline the importance of a careful bariatric follow-up including monitoring for HP infection.

Limitations of this study are its retrospective nature which opens it to possible selection bias, the impossibility to measure ghrelin and leptin serum levels and the relatively small number of recruited patients which can expose to the risk of a type II error. Another limitation is the relatively short follow-up period of these patients. Prospective, multi-centered, and long-term follow-up research studies are needed.

## 6 Conclusion

This study is a further contribution to understanding the complex relationship between chronic gastritis and *H. pylori* infection in patients undergoing LSG for obesity, highlighting the importance of routine evaluation of the gastric specimens and HP test after LSG, despite the negative preoperative gastroscopy. However, the possible role of chronic gastritis and HP infection in the prevention of an adequate weight loss after LSG still needs to be investigated further since the difference in postoperative weight loss in patients with and without chronic gastritis in our study did not reach statistical significance, suggesting the need for further prospective studies on a larger sample of patients and correlating data with ghrelin and leptin serum level.

## Abbreviations

BMI: body mass index
CG: chronic gastritis
EGD: esophagogastroduodenoscopy
IM: intestinal metaplasia
HP: *Helicobacter pylori*
LSG: laparoscopic sleeve gastrectomy
NoCG: absence of chronic gastritis %
EWL: excess weight loss
% EBL: excess BMI loss
